# Social Determinants of Health–Related Needs During COVID-19 Among Low-Income Households With Children

**DOI:** 10.5888/pcd17.200322

**Published:** 2020-10-01

**Authors:** Shreela V. Sharma, Ru-Jye Chuang, Melinda Rushing, Brittni Naylor, Nalini Ranjit, Mike Pomeroy, Christine Markham

**Affiliations:** 1 Department of Epidemiology, Human Genetics and Environmental Sciences, Michael & Susan Dell Center for Healthy Living, The University of Texas Health Science Center at Houston School of Public Health, Houston, Texas; 2 Department of Health Promotion and Behavioral Sciences, Michael & Susan Dell Center for Healthy Living, The University of Texas Health Science Center, Austin Regional Campus, Austin, Texas; 3 Brighter Bites, Houston, Texas; 4 Department of Health Promotion and Behavioral Sciences. The University of Texas Health Science Center at Houston School of Public Health, Houston, Texas

## Abstract

**Introduction:**

Little is known about the social needs of low-income households with children during the coronavirus-2019 (COVID-19) pandemic. Our objective was to conduct a cross-sectional quantitative and qualitative descriptive analysis of a rapid-response survey among low-income households with children on social needs, COVID-19–related concerns, and diet-related behaviors.

**Methods:**

We distributed an electronic survey in April 2020 to 16,435 families in 4 geographic areas, and 1,048 responded. The survey asked families enrolled in a coordinated school-based nutrition program about their social needs, COVID-19–related concerns, food insecurity, and diet-related behaviors during the pandemic. An open-ended question asked about their greatest concern. We calculated descriptive statistics stratified by location and race/ethnicity. We used thematic analysis and an inductive approach to examine the open-ended comments.

**Results:**

More than 80% of survey respondents were familiar with COVID-19 and were concerned about infection. Overall, 76.3% reported concerns about financial stability, 42.5% about employment, 69.4% about food availability, 31.0% about housing stability, and 35.9% about health care access. Overall, 93.5% of respondents reported being food insecure, a 22-percentage-point increase since fall 2019. Also, 41.4% reported a decrease in fruit and vegetable intake because of COVID-19. Frequency of grocery shopping decreased and food pantry usage increased. Qualitative assessment identified 4 main themes: 1) fear of contracting COVID-19, 2) disruption of employment status, 3) financial hardship, and 4) exacerbated food insecurity.

**Conclusion:**

Our study highlights the compounding effect of the COVID-19 pandemic on households with children across the spectrum of social needs.

**Summary**

**What is already known on this topic?**

Little is known about the social needs of low-income households with children during the coronavirus-2019 (COVID-19) pandemic.

**What is added by this report?**

We conducted a rapid-response survey on social needs, COVID-19–related concerns, and diet-related behaviors during the shelter-in-place phase of the US pandemic among low-income households with children enrolled in a nutrition program. We found high levels of financial instability; concern about unemployment, food insecurity, and COVID-19; and reduced frequency of eating out and grocery shopping.

**What are the implications for public health practice?**

Government and community agencies must have a role in developing short- and long-term strategies to address the needs of our most vulnerable and underserved populations.

## Introduction

Coronavirus disease 2019 (COVID-19), the disease caused by severe acute respiratory syndrome coronavirus 2 (SARS-CoV-2), is a worldwide pandemic (1). As of September 2020, the United States is the epicenter of the pandemic, with more than 6.3 million confirmed cases and more than 189,000 fatalities (2). Starting in early and mid-March, many US states began implementing social distancing measures and lockdowns, which prompted retail outlets, restaurants, schools, universities, businesses, and other entities to close, and implemented a work-from-home policy where possible. As a result of the pandemic, unemployment rose to an all-time high of 14.7% in April 2020, and more than 10% of Americans filed for unemployment from April through July 2020 (3). Furthermore, thousands of people diagnosed and hospitalized with COVID-19 are experiencing employment challenges in conjunction with large medical bills (4–6). For most Americans, access to health care is directly related to employment, so that loss of employment means loss of health care. A secondary effect of school closures is the lack of access to national school lunch and/or breakfast programs (free or reduced-price meals) for children, thus increasing the risk of food insecurity among low-income families. By April 2020, approximately 38% of American households were experiencing food insecurity (7). With the continued threat of COVID-19, unemployment and financial instability may rise, pushing families into making trade-offs between basic needs such as food and shelter (ie, paying rent this week instead of buying food) (8). Basic human needs, also called the social determinants of health, include employment, food security, housing security, access to health care, and transportation; the lack of these is linked to poor health outcomes (9).

Brighter Bites is a nonprofit, evidence-based school health program that distributes fresh produce weekly to low-income families and provides nutrition education in school and for parents (10,11). Only schools that have more than 75% of children enrolled in a free or reduced-price meals program are eligible to participate in Brighter Bites. The program, which uses a school co-op concept, is disseminated in Southwest Florida and 5 US cities: Houston, Austin, Dallas, New York City, and Washington, DC. More than 100 schools participated during the 2019–2020 school year. Brighter Bites has improved dietary behaviors and the home nutrition environment among participating families (10). However, as a result of COVID-19–related school closures, program implementation has paused. To better understand the ongoing needs of families and provide critical services during the pandemic, Brighter Bites conducted a rapid-response survey in April 2020 in 4 locations (Houston, Dallas, Washington, DC, and Southwest Florida) among participating families. The survey asked about financial stability, employment status, food security, housing security, transportation needs, access to child care, and access to health care. At the time the survey was conducted, all 4 locations were under shelter-in-place orders, which included social distancing, school closures, and suspension of all nonessential services. The objective of our study was to quantitatively and qualitatively describe the data obtained from this rapid-response survey.

## Methods

Brighter Bites distributed the survey electronically in April 2020 in English and Spanish via Formsite (Vroman Systems, Inc) to 16,436 Brighter Bites families (56.9% in Houston, 29.2% in Dallas, 7.8% in the District of Columbia, and 6.1% in Southwest Florida) who were enrolled in the program during the 2019–2020 school year and provided their telephone number in fall 2019. Program staff members distributed survey links through text messages. The overall survey response rate was 6.4% (1,048 of 16,436). Response rates by location were 7.7%, Houston; 3.8%, Dallas; 6.4%, Washington, DC; and 6.9%, Southwest Florida. Survey completion was voluntary. We obtained informed consent from all respondents, and one parent or adult per family completed the survey. Brighter Bites collected all data and shared de-identified data with the University of Texas Health Science Center for analysis as part of a data-sharing agreement. All procedures were approved by the University of Texas Health Science Center’s Committee for Protection of Human Subjects.

### Data collection

The 30-item self-report survey took approximately 10 minutes to complete.

**Sociodemographic characteristics.** The survey collected data on respondent’s sex, respondent’s relationship to the child, respondent’s and child’s race/ethnicity, respondent’s employment status, respondent’s education level, and enrollment in government assistance programs. Program options were the Special Supplemental Nutrition Program for Women, Infants, and Children (WIC), Supplemental Nutrition Assistance Program (SNAP), Double Dollars, Medicaid/Texas Health Steps, Medicare, national school lunch and/or breakfast programs (free or reduced-price meals), and Children’s Health Insurance Program (CHIP).

**COVID-19–related concerns.** The survey asked respondents how much they had seen or heard about COVID-19. Four response options ranged from “nothing at all” to “a great deal.” The survey also asked about respondents’ perceived concern for themselves and their children in contracting COVID-19. Response options were on a 4-point Likert-type scale ranging from strongly disagree to strongly agree. These questions were adapted from previously administered surveys (12).

**Social determinants of health.** The survey asked respondents about their concerns during the pandemic about financial stability, employment status, availability of food, affordability of food, availability or affordability of housing, access to reliable transportation, access to childcare, and access to a clinic or physician. The survey provided a checklist for these 8 items, and respondents could check all that applied. The survey also invited respondents to write in any other concerns by using this statement: “Please share your greatest concern at this time, or any other thoughts you would like to share with us.”

**Food insecurity.** The parent or another adult in the family used the 2-item Hunger Vital Sign screening questionnaire developed and validated by Hager et al (13) to report household food security status during the COVID-19 pandemic. If the respondent indicated “often true” or “sometimes true” to either of the 2 questions, we considered the household food insecure. If a respondent answered “never true” to both questions, we considered the household food secure.

**Food shopping frequency and behavior.** The survey used items adapted from the National Cancer Institute’s 2007 Food Attitudes and Behavior Survey (14). Respondents reported the frequency (times per month) and type of store at which their household shopped for fruits and vegetables. Response options were a large chain grocery store or supermarket, a small local store or corner store, a farmers market/food co-op/farm stand, and a food bank/food pantry or other food distributions. Additionally, the survey asked respondents about their store-shopping practices: whether they physically shopped inside the store, shopped online with curbside pick-up, or shopped online with goods delivered to home. Respondents could check all that applied.

**Dietary habits.** The survey asked respondents about their frequency of eating out at restaurants in the previous 7 days and whether their frequency of eating out had changed because of the pandemic (15). The survey also asked if, because of COVID-19, their consumption of fruits and vegetables had increased, decreased, or stayed the same.

### Statistical analysis

**Quantitative data analysis.** We performed all analyses on de-identified data using Stata version 15.0 (StataCorp LLC). We computed means, standard deviations, and frequency distributions overall and stratified by race/ethnicity. We used the χ^2^ test or Fisher exact test and 1-way analysis of variance to compute differences in responses by city and race/ethnicity. For 3 variables (food insecurity, frequency of eating out, and frequency of shopping for produce) we compared responses on similar items in data collected from 3,880 families in the same 4 locations in fall 2019, before the start of the Brighter Bites program; we used the χ^2^ test to assess changes. Significance was set at *P* <.05 for all tests. The fall 2019 survey and April 2020 survey used similar questions for the 3 variables, except for the question on type of store. Both surveys provided at least 1 example of each type of store, but the baseline survey did not list “other food distributions” in the same category as food bank/food pantry.

**Qualitative data.** We collated and analyzed responses to the open-ended question, “Please share your greatest concern at this time, or any other thoughts you would like to share with us.” We used thematic analysis to analyze the survey data by using an inductive approach in which we derived codes and themes on the basis of the content from the survey data (16,17). Two trained coders independently coded the comments by using an open coding method in Microsoft Word to establish initial coding themes and subthemes. Next, the 2 coders collectively assessed the themes and subthemes for agreement and created a codebook with definitions for each theme. Finally, the coders used this codebook to reconfirm the coding of the themes and subthemes in qualitative comments.

## Results

Overall, the demographic characteristics of respondents did not vary substantially by site (Table 1). Most (68.2%) respondents resided in Houston; 97.0% were female; 85.9% were Hispanic, 7.1% were African American, and 50.5% were mostly or only Spanish speaking; the mean age was 36.7 years. On average, 25.1% and 28.2% of families reported receiving WIC or SNAP, respectively. Also, 46.7% of families reported receiving Medicaid, and 74.4% of the children participated in free or reduced-price meals programs. A greater proportion in Houston and Washington, DC, than in the other 2 locations received Medicaid or CHIP (*P* < .001), whereas a smaller proportion of children in Dallas than in the other 3 locations participated in free or reduced-price meals programs (*P* = .04). These differences, however, were small.

**Table1:** Demographic Characteristics of Brighter Bite Respondents (N = 1,048) to a COVID-19 Survey of Low-Income Households With Children, Overall and by Location, April 2020^a^

Variable	Overall (N = 1,048)^b^	Houston (n = 715)^b^	Dallas (n = 181)^b^	Washington, DC (n = 82)^b^	Southwest Florida (n = 70)^b^	*P* Value^c^
**Percentage of respondents**	100.0	68.2	17.3	7.8	6.7	—
**Respondent race**						
Black or African American	74 (7.1)	52 (7.3)	7 (3.9)	12 (14.8)	3 (4.3)	.08^d^
Mexican American, Latino, or Hispanic	894 (85.9)	603 (85.1)	164 (90.6)	63 (77.8)	64 (91.4)	
Non-Hispanic White	38 (3.7)	29 (4.1)	6 (3.3)	1 (1.2)	2 (2.9)	
Other	35 (3.4)	25 (3.5)	4 (2.2)	5 (6.2)	1 (1.4)	
**Language spoken at home**						
Most or only English	169 (16.1)	124 (17.4)	23 (12.7)	12 (14.6)	10 (14.3)	.11^e^
Both English and Spanish equally	326 (31.1)	218 (30.5)	58 (32.0)	20 (24.4)	30 (42.9)	
Most or only Spanish	529 (50.5)	356 (49.9)	99 (54.7)	46 (56.1)	28 (40.0)	
Other language	23 (2.2)	16 (2.2)	1 (0.6)	4 (4.9)	2 (2.9)	
**Respondent sex**						
Male	31 (3.0)	18 (2.5)	7 (3.9)	2 (2.4)	4 (5.7)	.34^d^
Female	1,014 (97.0)	695 (97.5)	173 (96.1)	80 (97.6)	66 (94.3)	
**No. of people living in home, mean (SD)**						
Children (<18 y)	2.7 (1.1)	2.7 (1.1)	2.6 (1.1)	NA	2.7 (1.1)	.74^f^
Elders (≥65 y)	0.3 (0.6)	0.3 (0.6)	0.2 (0.5)	NA	0.05 (0.3)	.01^f^^,^ g
Adults (18 to <65 y)	2.4 (0.9)	2.4 (0.9)	2.5 (0.9)	NA	2.3 (1.0)	.44^f^
Total	4.7 (2.0)	5.1 (1.5)	5.1 (1.5)	NA	5.1 (1.2)	<.001^f^^,^ g
**Parent age, mean (SD), y**	36.7 (7.3)	36.8 (7.4)	36.4 (7.3)	NA	36.1 (6.3)	.64^f^
**Does your family use the following?**						
WIC	261 (25.1)	171 (24.0)	41 (23.0)	23 (28.8)	26 (37.1)	.08^d^
SNAP	293 (28.2)	213 (29.8)	46 (25.7)	19 (24.4)	15 (21.7)	.35^d^
Double Dollars	8 (0.8)	5 (0.7)	3 (1.7)	0	0	.43^d^
Medicaid/Texas Health Steps	485 (46.7)	372 (52.2)	84 (47.2)	11 (14.1)	18 (26.1)	<.001^d^^,^ g
Medicare	128 (12.3)	65 (9.1)	19 (10.7)	28 (35.0)	16 (22.9)	<.001^d^^,^ g
Free/reduced-price meals	777 (74.4)	543 (76.1)	118 (65.6)	62 (75.6)	54 (78.3)	.04^d^^,^ g
CHIP	173 (16.6)	100 (14.0)	35 (19.6)	29 (35.8)	9 (13.0)	<.001^d^^,^ g

Abbreviations: CHIP, Children’s Health Insurance Program; NA, not applicable; SNAP, Supplemental Nutrition Assistance Program; WIC, Special Supplemental Nutrition Program for Women, Infants, and Children.

a Brighter Bites is a nonprofit organization whose mission is to improve access to fresh fruits and vegetables and nutrition education among underserved communities. The survey was administered electronically in April 2020 to 16,436 Brighter Bites families who were enrolled in the program during the 2019–2020 school year. Response was voluntary. All values are number (percentage) unless otherwise indicated. Percentages may not add to 100 because of rounding.

b Number is the total number of survey responses received overall and in each city. Because survey completion was voluntary, not all respondents answered all questions; thus, values for each variable may not sum to value in column head.

c Significance set at *P* < .05 for all tests.

d Fisher exact test.

e χ^2^ test.

f 1-way analysis of variance.

g Stratified analysis showed significant differences (*P* < .05) in sociodemographic variables by city.

Because demographic characteristics did not vary by site, our examination of COVID-19–related knowledge, risks, attitudes, and behaviors across racial/ethnic groups did not adjust for site differences. Most (81.8%) respondents reported knowing a great deal about COVID-19 and were concerned about being infected themselves (87.3%) or their children being infected (87.0%) (Table 2). A smaller proportion of African American respondents than respondents in other racial/ethnic groups were concerned about being infected with COVID-19 themselves (38.7%) and about their child being infected (39.3%) (*P* < .001).

**Table 2:** Descriptive Analysis of COVID-19–Related Concerns, Social Determinants of Health, and Dietary Behaviors Among Brighter Bite Respondents (N = 1,048) to COVID-19 Survey of Low-Income Households With Children, Overall and by Race/Ethnicity, April 2020^a^

Variable	Overall (N = 1,048)^b^	Hispanic (n = 894)^b^	African American (n = 74)^b^	Non-Hispanic White (n = 38)^b^	Other (n = 35)^b^	*P* Value^c^
**Sociodemographic Characteristics**						
**Parent sex**						
Male	31 (3.0)	20 (2.2)	3 (4.1)	2 (5.4)	6 (17.1)	<.001^d^^,^ e
Female	1,008 (97.0)	873 (97.8)	71 (95.9)	35 (94.6)	29 (82.9)	
**Language spoken at home**						
Most or only English	169 (16.2)	68 (7.6)	61 (82.4)	26 (68.4)	14 (40.0)	<.001^d^^,^ e
Both English and Spanish equally	326 (31.3)	314 (35.1)	3 (4.1)	8 (21.1)	1 (2.9)	
Most or only Spanish	524 (50.3)	508 (56.8)	2 (2.7)	3 (7.9)	11 (31.4)	
Other language	22 (2.1)	4 (0.5)	8 (10.8)	1 (2.6)	9 (25.7)	
**No. of people living in home**						
Children (<18 y)	2.7 (1.1)	2.7 (1.1)	2.7 (1.1)	2.6 (1.2)	2.3 (1.0)	.38^f^
Elders (≥65 y)	0.2 (0.6)	0.2 (0.5)	0.5 (0.8)	0.4 (0.8)	0.5 (0.8)	.005^e^^,^ f
Adults (18 to <65 y)	2.4 (0.9)	2.4 (0.9)	2.0 (0.9)	2.2 (0.9)	2.1 (0.8)	.001^e^^,^ f
Total	4.8 (1.1)	4.8 (1.9)	4.1 (2.2)	4.8 (1.7)	4.2 (2.2)	.004^e^^,^ f
**Parent age, mean (SD), y**	36.6 (7.2)	36.2 (6.7)	40.3 (10.6)	38.4 (9.6)	38.2 (7.3)	<.001^e^^,^ f
**Does your family use the following?**						
WIC	258 (24.9)	214 (24.1)	26 (35.1)	9 (23.7)	9 (25.7)	.21^f^
SNAP	290 (28.1)	241 (27.2)	27 (36.5)	13 (34.2)	9 (25.7)	.29^f^
Double Dollars	8 (0.8)	5 (0.6)	2 (2.7)	1 (2.6)	0	.10^d^
Medicaid/Texas Health Steps	481 (46.6)	406 (45.9)	42 (56.8)	20 (52.6)	13 (37.1)	.17^f^
Medicare	127 (12.3)	102 (11.5)	14 (18.9)	8 (21.1)	3 (8.6)	.08^d^
Free or reduced-price meals	771 (74.2)	661 (74.1)	60 (81.1)	28 (73.7)	22 (62.9)	.24^f^
CHIP	172 (16.6)	148 (16.7)	14 (18.9)	4 (10.5)	6 (17.1)	.75^d^
**COVID-19–Related Concerns**						
**How much have you seen or heard about COVID-19?**						
Nothing at all	3 (0.3)	2 (0.2)	1 (1.4)	0	0	<.001^d^^,^ e
A fair amount	60 (5.8)	42 (4.7)	6 (8.1)	5 (13.2)	7 (20.0)	
Not very much	127 (12.2)	120 (13.4)	3 (4.1)	2 (5.3)	2 (5.7)	
A great deal	851 (81.8)	730 (81.7)	64 (86.5)	31 (81.6)	26 (74.3)	
**I am concerned that I will get the coronavirus**						
Strongly disagree	37 (3.9)	12 (1.5)	17 (27.4)	6 (16.2)	2 (6.3)	<.001^d^^,^ e
Disagree	82 (8.7)	51 (6.3)	21 (33.9)	7 (18.9)	3 (9.4)	
Agree	247 (26.3)	211 (26.2)	11 (17.7)	12 (32.4)	13 (40.6)	
Strongly agree	572 (61.0)	533 (66.1)	13 (21.0)	12 (32.4)	14 (43.8)	
**I am concerned that my child will get the coronavirus (question asked of parents only)**						
Strongly disagree	47 (4.9)	22 (2.6)	18 (29.5)	5 (13.5)	2 (6.1)	<.001^d^^,^ e
Disagree	78 (8.1)	47 (5.7)	19 (31.2)	8 (21.6)	4 (12.1)	
Agree	208 (21.6)	176 (21.3)	13 (21.3)	13 (35.1)	5 (15.2)	
Strongly agree	631 (65.4)	582 (70.4)	11 (18.0)	11 (29.8)	22 (66.7)	
**Due to the coronavirus, are you concerned about any of the following in regards to you and your family? (check all that apply)**						
Financial stability	794 (76.3)	682 (76.3)	58 (78.4)	30 (78.9)	24 (68.6)	.69^g^
My employment status will change in the near future	442 (42.5)	377 (42.2)	29 (39.2)	17 (44.7)	19 (54.3)	.49^g^
Availability of food	722 (69.4)	612 (68.5)	51 (68.9)	30 (78.9)	29 (82.9)	.17^g^
Affordability of food	515 (49.5)	424 (47.4)	48 (64.9)	24 (63.2)	19 (54.3)	.009^e^^,^ g
Availability or affordability of housing	323 (31.0)	264 (29.5)	31 (41.9)	13 (34.2)	15 (42.9)	.06^g^
Access to reliable transportation	67 (6.4)	57 (6.4)	5 (6.8)	2 (5.3)	3 (8.6)	.88^d^
Access to childcare	85 (8.2)	56 (6.3)	17 (23.0)	6 (15.8)	6 (17.1)	<.001^d^^,^ e
Access to your clinic/doctor	374 (35.9)	314 (35.1)	31 (41.9)	16 (42.1)	13 (37.1)	.56^d^
**Which of the following is true for you?**						
I have not experienced any symptoms of coronavirus	1,033 (99.2)	888 (99.3)	73 (98.6)	37 (97.4)	35 (100.0)	.36^d^
I am currently experiencing the symptoms but have not been diagnosed	6 (0.6)	4 (0.5)	1 (1.4)	1 (2.6)	0	
I have already been diagnosed with coronavirus	2 (0.2)	2 (0.2)	0	0	0	
I have been diagnosed with coronavirus and have recovered	0	0	0	0	0	
**Prevalence of Preexisting Health Conditions Known to Increase Risk of COVID-19**						
**Check any of the below health conditions apply to you or a member of your immediate family who live with you**						
**Diagnosed by a doctor as having diabetes**						
No one in my family	700 (74.5)	609 (74.6)	48 (80.0)	26 (72.2)	17 (60.7)	.40^d^
Myself	58 (6.2)	51 (6.3)	4 (6.7)	2 (5.6)	1 (3.6)	
One or more member(s) of my family	182 (19.4)	156 (19.1)	8 (13.3)	8 (22.2)	10 (35.7)	
**Diagnosed by a doctor as having heart disease**						
No one in my family	854 (90.9)	742 (90.9)	59 (98.3)	29 (80.6)	24 (85.7)	.05^d^
Myself	14 (1.5)	14 (1.7)	0	0	0	
One or more member(s) of my family	72 (7.7)	60 (7.4)	1 (1.7)	7 (19.4)	4 (14.3)	
**Diagnosed by a doctor as having autoimmune conditions or going through cancer treatment**						
No one in my family	862 (91.6)	757 (92.5)	52 (88.1)	30 (83.3)	23 (82.1)	.007^d^^,^ e
Myself	25 (2.7)	16 (2.0)	5 (8.5)	2 (5.6)	2 (7.1)	
One or more member(s) of my family	54 (5.7)	45 (5.5)	2 (3.4)	4 (11.1)	3 (10.7)	
**Diagnosed by a doctor as having chronic lung disease or moderate to severe asthma**						
No one in my family	772 (81.8)	673 (82.1)	51 (85.0)	25 (69.4)	23 (82.1)	.10^d^
Myself	25 (2.7)	18 (2.2)	3 (5.0)	3 (8.3)	1 (3.6)	
One or more member(s) of my family	147 (15.6)	129 (15.7)	6 (10.0)	8 (22.2)	4 (14.3)	
**Is a current smoker**						
No one in my family	860 (90.7)	763 (92.6)	49 (81.7)	25 (69.4)	23 (82.1)	<.001^d^^,^ e
Myself	21 (2.2)	12 (1.5)	4 (6.7)	4 (11.1)	1 (3.6)	
One or more member(s) of my family	67 (7.1)	49 (6.0)	7 (11.7)	7 (19.4)	4 (14.3)	
**How would you rate your current health status?**						
Poor	26 (2.4)	22 (2.5)	2 (2.7)	0	1 (2.9)	.64^d^
Fair	235 (22.6)	203 (22.7)	12 (16.2)	11 (28.9)	9 (25.7)	
Good	469 (45.1)	405 (45.4)	31 (41.9)	16 (42.1)	17 (48.6)	
Very good	183 (17.6)	154 (17.3)	16 (21.6)	7 (18.4)	6 (17.1)	
Excellent	128 (12.3)	109 (12.2)	13 (17.6)	4 (10.5)	2 (5.7)	
**Food Insecurity and Diet-Related Behaviors During COVID-19 Pandemic**						
**Food insecurity due to coronavirus**						
Food secure	68 (6.5)	47 (5.3)	8 (10.8)	6 (15.8)	7 (20.0)	<.001^g^
Food insecure	973 (93.5)	847 (94.7)	66 (89.2)	32 (84.2)	28 (80.0)	
**During the past 7 days, how many times did your family eat food from any type of restaurant (including take-out)?**						
Never	641 (61.6)	561 (62.8)	45 (60.8)	13 (34.2)	22 (62.9)	<.001^d^^,^ e
1–2 times per week	370 (35.6)	317 (35.5)	22 (29.7)	21 (55.3)	10 (28.6)	
3–4 times per week	21 (2.0)	12 (1.3)	5 (6.8)	3 (7.9)	1 (2.9)	
5–6 times per week	6 (0.6)	3 (0.3)	1 (1.4)	1 (2.6)	1 (2.9)	
≥7 times per week	2 (0.2)	0	1 (1.4)	0	1 (2.9)	
**Due to coronavirus, has your frequency of eating food from restaurants changed?**						
Decreased	869 (83.5)	759 (84.9)	55 (74.3)	26 (68.4)	29 (82.9)	.003^d^^,^ e
Stayed the same	133 (12.8)	105 (11.7)	17 (23)	6 (15.8)	5 (14.3)	
Increased	39 (3.8)	30 (3.4)	2 (2.7)	6 (15.8)	1 (2.9)	
**Due to coronavirus, currently how often do you buy or get fruits and vegetables and other groceries for the family from these locations?**						
**A large grocery store or super market?**						
Never	30 (2.9)	28 (3.1)	0	1 (2.6)	1 (2.9)	.02^d^^,^ e
Less than once a month	130 (12.5)	103 (11.5)	16 (21.6)	2 (5.3)	9 (25.7)	
1 or 2 times per month	373 (35.8)	330 (36.9)	22 (29.7)	14 (36.8)	7 (20.0)	
1 time per week	379 (36.4)	331 (37.0)	21 (28.4)	15 (39.5)	12 (34.3)	
≥2 times per week	129 (12.4)	102 (11.4)	15 (20.3)	6 (15.8)	6 (17.1)	
**A small local store or corner store**						
Never	485 (46.6)	409 (45.8)	32 (43.2)	21 (55.3)	23 (65.7)	.009^d^^,^ e
Less than once a month	177 (17.0)	145 (16.2)	20 (27.0)	8 (21.1)	4 (11.4)	
1 or 2 times per month	171 (16.4)	150 (16.8)	12 (16.2)	2 (5.3)	7 (20.0)	
1 time per week	161 (15.5)	150 (16.8)	4 (5.4)	7 (18.4)	0	
≥2 times per week	46 (4.4)	39 (4.4)	6 (8.1)	0	1 (2.9)	
**A farmers market/food co-op/farm stand**						
Never	828 (82.7)	725 (84.7)	45 (61.6)	30 (79.0)	28 (82.4)	<.001^d^^,^ e
Less than once per month	87 (8.7)	65 (7.6)	14 (19.2)	4 (10.5)	4 (11.8)	
1 or 2 times per month	42 (4.2)	29 (3.4)	8 (11.0)	3 (7.9)	2 (5.9)	
1 time per week	27 (2.7)	23 (2.7)	3 (4.1)	1 (2.6)	0	
≥2 times per week	17 (1.7)	14 (1.6)	3 (4.1)	0	0	
**A food bank/food pantry, or other food distributions**						
Never	516 (49.7)	448 (50.2)	31 (41.9)	18 (47.4)	19 (54.3)	.12^d^
Less than once a month	214 (20.6)	179 (20.1)	24 (32.4)	7 (18.4)	4 (11.4)	
1 or 2 times per month	169 (16.3)	138 (15.5)	14 (18.9)	11 (29.0)	6 (17.1)	
1 time per week	114 (11.0)	103 (11.6)	4 (5.4)	2 (5.3)	5 (14.3)	
≥2 times per week	26 (2.5)	24 (2.7)	1 (1.4)	0	1 (2.9)	
**At this time, how do you or your family member(s) shop at these grocery stores or super markets? (check all that apply)**						
Physically shop inside the store	970 (93.2)	834 (93.3)	71 (96.0)	35 (92.1)	30 (85.7)	.24^d^
Shop online with curbside pick-up	79 (7.6)	56 (6.3)	13 (17.6)	7 (18.4)	3 (8.6)	.001^d^^,^ e
Shop online and delivered to home	21 (2.0)	11 (1.2)	6 (8.1)	0	4 (11.4)	<.001^d^^,^ e
**Due to the coronavirus, has your consumption of fruits and vegetables . . . **						
Decreased	431 (41.4)	367 (41.1)	27 (36.5)	18 (47.4)	19 (54.3)	.57^g^
Stayed the same	296 (28.4)	260 (29.1)	21 (28.4)	8 (21.1)	7 (20.0)	
Increased	314 (30.2)	267 (29.9)	26 (35.1)	12 (31.6)	9 (25.7)	

a Brighter Bites is a nonprofit organization whose mission is to improve access to fresh fruits and vegetables and nutrition education among underserved communities. The survey was administered the survey electronically in April 2020 to 16,436 Brighter Bites families who were enrolled in the program during the 2019–2020 school year. Response was voluntary. All values are number (percentage) unless otherwise indicated. Percentages may not add to 100 because of rounding.

b Number is the total number of survey responses received overall and by race/ethnicity. Because survey completion was voluntary, not all respondents answered all questions; thus, values for each variable may not sum to value in column head.

c Significance set at *P* < .05 for all tests.

d Fisher exact test.

e Stratified analysis showed significant differences (*P* < .05) in sociodemographic variables by city.

f 1-way analysis of variance.

g χ^2^ test.

Overall, 76.3% of respondents reported concerns about financial stability, 42.5% were concerned about their employment, 69.4% about availability of food, 49.5% about affordability of food, 31.0% about housing stability, and 35.9% about access to a clinic or physician. A small proportion of respondents expressed concern about reliable transportation (6.4%) and childcare (8.2%). When we stratified results by race/ethnicity, a significantly greater proportion of African American (64.9%) and Non-Hispanic White (63.2%) respondents, compared with Hispanic respondents or in the “other” racial/ethnic group, was concerned about affordability of food (*P* = .009). Moreover, a smaller proportion of Hispanic respondents (6.3%) than African American (23.0%), Non-Hispanic White (15.8%), or “other” (17.1%) racial ethnic groups was concerned with access to childcare (*P* < .001) (Table 2).

Overall, diagnosis of COVID-19 and prevalence of pre-existing conditions, such as diabetes, heart disease, and being immune compromised, were less than 5% among respondents at the time of survey completion. When asked about their general health status, 25.0% of respondents reported their health status to be fair or poor, with no significant differences by race/ethnicity.

Overall, 93.5% of respondents reported being food insecure in April 2020. In fall 2019, before Brighter Bites, 71.5% of respondents reported being food insecure (*P* < .001) (Figure). A significantly greater proportion of Hispanic respondents (94.7%) than respondents in other racial/ethnic groups reported food insecurity (*P* < .001) (Table 2). Overall, 61.6% of respondents reported never eating food from a restaurant during the previous 7 days (including take-out), with a smaller proportion of non-Hispanic White families (34.2%) than other racial/ethnic groups never eating out (*P* < .001). In fall 2019, 12.7% of families reported never eating out (*P* < .001). In addition, 83.5% of respondents reported decreasing the frequency of eating out because of COVID-19, with a greater proportion of Hispanic families (84.9%) than families in other racial/ethnic groups reporting decreased frequency (*P* = .003).

**Figure:**
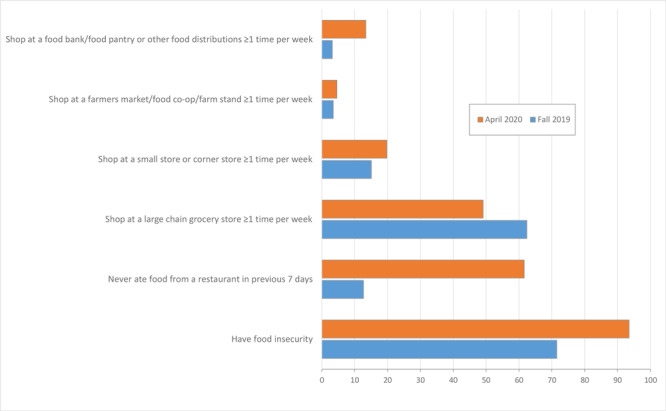
COVID-19-related changes in food insecurity, eating out, and food shopping behavior among low-income households with children (N = 1,048), Brighter Bites. All changes were significant at *P* < .001, except for shopping at a farmers market/food co-op/farm stand ≥1 time per week (*P* = .31).

**Table:** 

Factor	Fall 2019, %	April 2020, %	*P* value
Have food insecurity	71.5	93.5	<.001
Never ate food from a restaurant in previous 7 days	12.7	61.6	<.001
Shop at a large chain grocery store ≥1 time per week	62.4	49.1	<.001
Shop at a small store or corner store ≥1 time per week	15.1	19.8	<.001
Shop at a farmers market/food co-op/farm stand ≥1 time per week	3.5	4.6	.31
Shop at a food bank/food pantry or other food distributions ≥1 time per week	3.2	13.4	<.001

Most (97.1%) Brighter Bites families reported shopping at a large grocery store during the pandemic but at varying frequencies. Almost half (49.1%) reported in April 2020 shopping at least once per week, whereas 62.4% reported shopping at least once per week in fall 2019 (*P* < .001) (Figure). Moreover, in fall 2019 only 3.2% reported shopping at a food bank or food pantry or at other distributions at least once per week, where 13.4% reported shopping at these food outlets at least once per week in April 2020 (*P* < .001). When asked about the effect of COVID-19 on fruit and vegetable consumption, 41.4% of families reported a decrease in intake of fruits and vegetables because of COVID-19; we found no significant differences by race/ethnicity in this response (Table 2).

### Qualitative analysis

Four themes emerged from the analysis of write-in responses to the question about immediate concerns during the COVID-19 pandemic (Table 3): 1) fear of contracting COVID-19, 2) disruption of employment status, 3) financial hardship, and 4) exacerbated food insecurity. Collectively, all themes stemmed from fear of contracting COVID-19. As indicated in subthemes and comments, many respondents were concerned about contracting COVID-19 themselves or concerned about a family member contracting COVID-19. The concerns were due to various reasons, such as lack of personal protective equipment and working in frontline jobs. Respondents also expressed concern about an inability to pay bills, rent, and for other basic needs because of closure-related unemployment. Respondents were also uncertain about how they were going to access food as social distancing and the pandemic continued. In addition, respondents were concerned about limited produce at various food stores, spiked produce prices, and the finances required to purchase food for their families.

**Table 3:** Thematic Assessment of Comments From Low-Income Households With Children (N = 1,048), Brighter Bites COVID-19 Survey, April 2020

Themes	Description of Code	Quote
**Fear of contracting COVID-19**	Fear of contracting COVID-19 for family	My biggest worry is that my family gets infected. I worry that I get sick since I’m pregnant.
	Concerned about when COVID-19 will end	My worry is that this won’t end and we won’t be able to take care of our kids.
	Fear of COVID-19 spreading	I’m worried about people making each other sick since not everyone is practicing distancing and you don’t know who is sick because they haven’t checked everyone (like for example when you go to the supermarket and people aren’t wearing masks).
	Fear of children contracting COVID-19	My biggest worry is getting sick, or that my kids get sick, or ending up without food or money to pay the bills.
	Fear of going out (eg, stores) because of COVID-19	My biggest worry is going out and getting the coronavirus.
	Fear of grocery shopping because of COVID-19	A lot of fear about getting infected while going to the store early to buy food, and that my kids get sick.
	Fear of returning to normalcy after COVID-19	Going back to be in a store, park, schools and being with the family, without any worry of some infection.
	Fear of safety for family	We should stay home. I’m worried about my children’s safety.
	Lack of seriousness about COVID-19 among other people	A lot of people go to the supermarkets, Walmart, H.E.B, Fiesta, among others without masks, latex gloves and at the entrance of these establishments, nobody says anything to them, they also touch products and the carts without gloves and many parents take their kids to the supermarket.
	Taking necessary preventive measurements for family at home to avoid contracting COVID-19	Trying not to go out and follow the recommendation to stay at home and not expose kids to this virus.
	Concern about having to work with no personal protective equipment	I’m worried about my husband getting sick and spreading it to me and my kids because he’s still working and his job doesn’t provide protection.
	Fear because of COVID-19 of having to go into work	My biggest concern is about my husband who is working picking tomatoes and he says that a lot of the workers aren’t being as careful as they should about coronavirus and that the bosses don’t care about them. Because a lot of people already have the flu. And I’m scared that we’re all going to get sick.
**Disruption of employment status**	Lack of employment	What’s happening is really hard because there’s no work but being healthy is the important thing.
	Fear of being unemployed	My worry is that my husband can lose his job due to the pandemic that we’re going through.
	Working less hours due to COVID-19	That my husband has very little work.
	Lack of employment at a restaurant	My biggest worry is that this virus last for months since I work in a restaurant, and my savings are drying up day by day.
**Financial hardship**	Concern about not having enough money to pay bills/utilities	We’re just worried about how to pay the bills this month.
	Concern about not having enough money to pay rent	My biggest worry is not being able to pay next month’s rent and not knowing where to go.
	Lack of finances	I’m worried about my children’s food and about work. There are bills to pay and no money.
	Unable to meet kids’ needs	I’m worried about how to feed my kids because there’s no work right now.
	Fear of being homeless	That we don't have work and can’t pay the rent and bills, and that this ends up with my kids having no place to live.
	Fear of inability to take care of family	I am the only financial support for my family, so if anything happened to me, I don't know what would happen to my loved ones.
	Lack of necessities/supplies	That we don’t have masks, gloves, or disinfectant wipes because you can’t find them at the stores.
	Fear of not having necessities/ supplies	I’m worried that my kids won’t have food (fruits, vegetables, milk, and basic hygiene items) because there’s not enough opportunity for work.
	Economic instability	Economic instability, since unemployment comes from it.
	Financial instability	It’s stressful, and the financial uncertainty.
	Worry/fear of future financial challenges	My biggest worry is not having an income for my family and that our savings run out.
	Ineligible for government assistance	Food is running out for my children, I have no work, no money, I don’t even qualify for the check that Mr. Donald Trump is sending out.
	Reduced quality of life	We’re just worried about having a better standard of living than right now. I hope this is over fast. There’s no work, no money and bills don’t offer forgiveness. There’s no help with bills, and the food is very limited.
	Concern about increased prices of food in stores	That food is scarce, and when there is some the price is high, starting with our produce.
**Exacerbated food insecurity**	Concern about not having enough food for family and/or children	I’m worried about not having enough food for my kids.
	Lack of food for family and/or children	I don't have enough food for my kids.
	Need for produce	We need more fruits and vegetables.
	Immediate need for food	I feel worried because I’m not giving enough vegetables and fruit to my children since my husband only works 3 days, and I’m not working because my baby was just born, so there are 4 children and 2 adults and I’m short of food and diapers for my baby, but what I need is food for the family.
	Decreased amount of food	[T]he fact that they can’t eat enough fruit or food that they like.
	Fear of grocery shopping because of COVID-19	That I don’t want to go to the store and that I run out of food, scared to go out.
	Stores not having enough food for buyers	It’s been difficult to find fresh produce in the stores.
	Concern about increased prices of food in stores	That food is scarce, and when there is some the price is high, starting with our produce.
	Fear of stores closing and not being able to get food	That they close all the stores where you can find food.

## Discussion

The COVID-19 pandemic is occurring in the context of a global economic crisis, both of which highlight health and social challenges for the most vulnerable people in our communities. To our knowledge, our study is the first to use a mixed-methods qualitative and quantitative assessment to understand the social needs of low-income families with children during the pandemic. Moreover, our study provides insight into these needs during the pandemic’s acute phase, when all 4 cities in the study sample were under shelter-in-place orders.

Understanding the social needs of our most vulnerable families with children is critical because of the health disparities associated with COVID-19 prevention and treatment (18). Low-income racial/ethnic minority populations, predominantly Hispanic and African American populations, are struggling with an increased risk of COVID-19 infection and COVID-19–related complications and mortality (19–22). These increases in risk are likely due to health inequities, which result in a disproportionately greater prevalence of obesity, diabetes, respiratory disorders and other predisposing conditions among Hispanic and African American populations (23). Inequities exist in social and structural factors, such as access to food, employment, transportation, housing, poverty, education, and health literacy (24). An overwhelming majority of our study families were food insecure, were experiencing financial instability, and had concerns about their employment status. Furthermore, about one-third of respondents expressed concern about access to health care, which would increase their risk of illness of death, not just from COVID-19, but from other health conditions as well.

We obtained further insights into the needs and concerns of survey respondents in the qualitative comments, which identified 4 themes: fear of contracting COVID-19, unemployment, financial hardship, and food insecurity. Interestingly, a smaller proportion of African American respondents than respondents in other racial/ethnic groups were concerned with being infected with COVID-19. This difference may be due to a disparity in health literacy and should be explored in future studies, given the high proportion of COVID-19–related complications and death in the African American population. Our qualitative assessment also demonstrated that although most respondents reported following safety guidelines, some expressed fear of becoming infected in public spaces. One subtheme was lack of personal protective equipment among family members working in a high-risk environment. In light of these concerns, Brighter Bites has partnered with other nonprofit organizations to provide masks for families at many of their food distribution locations. Similarly, concerns about disruption of employment and financial hardship identified such acute problems as inability to pay rent or bills. These results underscore how COVID-19 and the related economic crisis have not only caused physical harm but have further destabilized people who were already struggling. The number of unemployed people in the United States increased by 11.5 million from February to September 2020 (25), and this number may increase. Brighter Bites developed a set of comprehensive resources to disseminate to their families via text, emailed newsletters, and the Brighter Bites website (www.brighterbites.org). These resources include information on where to get tested for COVID-19, how to register for government assistance programs, COVID-19 prevention practices, and maintaining healthy eating, physical activity, and mental health.

Our study demonstrated a significant increase in food insecurity during the pandemic and a decrease in intake of healthy food such as fruits and vegetables. In addition, 41% of respondents said that their intake of fruits and vegetables decreased because of COVID-19, and 83% said their frequency of eating food from restaurants decreased because COVID-19. Qualitative comments provided additional insight, with many respondents expressing a concern about a general scarcity of food. This general scarcity was attributed to a lack of availability and affordability and less frequent grocery shopping because of presumed store closures or fear of contracting COVID-19. Seligman et al posited a conceptual framework for the relationships among financial stress, food insecurity, and health (26). They suggested that the combination of stress (resulting from unemployment and financial hardship) and poor nutrition can challenge disease prevention and management because these stressors strain the household budget, leaving little money for healthy foods and resulting in disordered eating (ie, consumption of unhealthy processed, nutrient-deficient foods), and increased medical care resulting in spending trade-offs (26). Furthermore, school and childcare closures, which halt school meals for children, have further pushed families into stress and food insecurity. Strategies to support meals for children, such as expanding and supporting enrollment in government assistance programs (eg, Pandemic-EBT), distribution of food for pick-up through schools, and other policy strategies should be considered (27,28).

The strengths of our study include a sample drawn from 4 US locations and a survey administered when all 4 areas were experiencing similar COVID-19 mitigation strategies. In addition, the study fills a gap in knowledge on the social needs of low-income households with children during COVID-19. Moreover, we conducted the qualitative analysis to help contextualize the quantitative findings, and these analyses indicated COVID-19–related shifts in food-related practices and behaviors among respondents. 

Our study has several limitations. Our sample is not representative of the general population in the 4 locations surveyed or Brighter Bites members but is a biased sample of families who likely needed help during this time. Furthermore, the sample size differed across the locations on the basis of the proportion of families enrolled in the Brighter Bites program. Also, the changes we observed in food insecurity, eating out, and food shopping practices could be attributed to other policy, systems, or environmental factors unrelated to COVID-19. Finally, our cross-sectional descriptive study does not allow us to draw conclusions on cause and effect; our study is a snapshot at an early moment in the pandemic and serves as a baseline for examining patterns of change and causal associations in future studies.

Our results highlight the compounding effect of the COVID-19 pandemic across the spectrum of basic social needs among low-income households with children. Our study is a call to action for government and community agencies to deploy short- and long-term strategies to address the needs of our most vulnerable populations.
